# Can smartphone use affect chronic disease self-management among Chinese middle-aged and older adults? A moderated mediation model

**DOI:** 10.3389/fpsyg.2022.1019335

**Published:** 2022-12-22

**Authors:** Fangmin Gong, Zhaowen Lei, Hewei Min, Yebo Yu, Zhen Huang, Jingyao Liu, Wenyu Wu, Jingqi Tang, Xinying Sun, Yibo Wu

**Affiliations:** ^1^School of Literature and Journalism Communication, Jishou University, Jishou, China; ^2^School of Public Health, Peking University, Beijing, China; ^3^School of Public Health, Shandong University, Jinan, Shandong Province, China; ^4^School of Health Management, Harbin Medical University, Harbin, Heilongjiang Province, China; ^5^School of Philosophy, Anhui University, Hefei, Anhui Province, China

**Keywords:** family health, middle-aged and elder people, chronic disease self-management, moderated mediation model, frequency of smartphone use

## Abstract

**Introduction:**

Chronic disease self-management is influenced by many factors. Previous studies have linked patients’ media use with chronic disease self-management, but the underlying mechanisms of this relationship are less understood.

**Objectives:**

The purpose of this study is to explore the mediating role of family health (FH) between frequency of smartphone use (FOSU) and self-management behaviors among middle-aged and older patients with chronic diseases (SBAMAOPWCD) through a moderated mediation model, and whether this indirect relationship is modified by the solitary status of middle-aged and older Chinese patients with chronic disease.

**Methods:**

Surveys were collected from 1,424 (*N* = 1,424; age > 45) middle-aged and older with one or more chronic conditions in China on self-reports of FOSU, FH and Chronic disease self-management behaviors were used to examine the moderated mediation model.

**Results:**

The results showed that the FOSU was significantly and positively associated with SBAMAOPWCD (*β* = 0.220, *p* < 0.001; *β* = 0.170, *p* < 0.001; *β* = 0.167, *p* < 0.001; *β* = 0.158, *p* < 0.001); The Family health resources (FHR) dimension of FH and the Family external social supports (FESS) dimension mediated the relationship between the FOSU and SBAMAOPWCD (*β* = −0.0758, CI: −0.1402, −0.0236; *β* = 0.0721, CI: 0.0141, 0.1458), Among them, the FHR dimension mediated mainly among FOSU, exercise and cognitive symptom management practices (CSMP; *β* = −0.0344, CI: −0.0652, −0.0102; *β* = −0.0401, CI: −0.0725, −0.0138), the FESS dimension of the FH mediated the relationship between the FOSU and communication with physicians (CWP; *β* = 0.0376, CI: 0.0116, 0.0705); Solitary state played a moderating role in the relationship between FHR dimension and SBAMAOPWCD (live alone *β* = −0.2395, CI: −0.4574, −0.0661; not live-alone *β* = −0.0599, CI: −0.1164, −0.0172). In addition, solitary state played a moderating role in the relationship among FHR dimension and CSMP for middle-aged and older patients (live alone *β* = −0.1095, CI: −0.1961, −0.0378; not live-alone *β* = −0.0334, CI: −0.0633, −0.0102). Interestingly, the relationship between FESS dimension and SBAMAOPWCD was moderated only by the non-live alone population (*β* = 0.0676, CI: 0.0008, 0.1478), and not by the live-alone population (*β* = 0.1026, CI: −0.1061, 0.3278).Unexpectedly, we found that when their FHR were lower, they reported higher levels of chronic disease self-management, middle-aged and older patients with chronic diseases who live alone are more significant in this impact relationship.

**Conclusions:**

The study further deepens our understanding of the mechanisms linking frequency of smartphone use with chronic disease self-management behaviors, and it helps to develop interventions to improve chronic disease self-management behaviors in middle-aged and older adults.

## Introduction

With the aging of the population, the globalization of the economy and the increasing prevalence levels of risk factors for chronic diseases, the number of people suffering from chronic diseases is rising and poses a serious burden on human health (WHO, 2022). The burden of meeting the needs of an increasing number of people will fall on already overstretched health care services that are struggling to cope with the demands of acute care, not to mention the needs of those with long-term health conditions. This has led to a gradual shift in responsibility for day-to-day disease management from health care professionals to individuals (Barlow et al., 2002). Self-management has become an important part of the overall management of chronic diseases (Wagner et al., 1996). Self-management is the ability and process by which individuals manage symptoms, treatments, physical and psychosocial consequences, and health behavior change (Barlow et al., 2002). As self-management can improve social, psychological, functional and clinical outcomes associated with chronic disease and reduce the use of health services (Walker et al., 2003). Therefore, interventions in the chronic disease self-management behaviors of middle-aged and older adults with chronic diseases are needed to help them adopt and maintain long-term health behavior changes to prevent further disease progression and improve quality of life (Peterson et al., 2010). For people with chronic diseases themselves, it is important to learn skills and information to help them develop and maintain healthy lifestyle changes (Kim et al., 2020). Observational learning is humans’ capacity to “expand their knowledge and skills rapidly through information conveyed by the rich variety of models” (Bandura, 2001a). Due to the limited number of models in the individual’s direct environment, observational learning often occurs in mediated environments, facilitated by communication technologies that mediate communication, such as television, computers, and smartphones (Bandura, 2001b).

In China, chronic diseases account for 88.5% of all deaths (National Health Commission, 2020). China is a developing country with a large population, low *per capita* income and rapidly developing Internet technologies. As we explore the relationship between smartphones and chronic disease self-management behaviors among middle-aged and older Chinese patients with chronic diseases, we may, to some extent, have some policy implications for other developing countries. Smartphones and mobile internet have become an integral part of people’s daily life in China (China Internet Network Information Center, 2021). However, few empirical studies have been conducted in China to analyze the impact of smartphones on chronic disease self-management among middle-aged and older adults. Previous research has focused on other forms of ICT (e.g., the Internet in general, computers, and social networking sites; Heo et al., 2015; Khalaila and Vitman-Schorr, 2017; Quintana et al., 2018). But the mobile Internet may have a greater impact on the lives of older adults than the PC-based Internet. As Puspitasari and Ishii (2016) point out, mobile phones are cheaper and more familiar to the average person than computers, so even the information-poor (e.g., older, less educated, less affluent) can enjoy the benefits and convenience of mobile technology. Previous chronic disease self-management has had a number of problems, including difficulty engaging patients, physicians, and organizations; problems accessing and evaluating them; and, they have had limited success in terms of content, time, and economic and social impact, and one economically and socially viable solution to alleviate some of these problems is to leverage the benefits of modern mobile technology (e.g., smartphones; Azevedo et al., 2015). A randomized controlled study by Bao et al. (2022) found mHealth intervention for TB self-management could improve their objective initiative and self-care management behaviors (Bao et al., 2022).

Although previous studies have linked mobile technology interventions to chronic disease self-management, many have focused more on the technological problem solving for chronic disease self-management and monitoring in middle-aged and older adults (Doyle et al., 2021; O’Reilly et al., 2022).Less learned about the mechanisms and mediators underlying the use of mobile technology to intervene in the self-management behaviors of middle-aged and older patients with chronic diseases. The mechanisms and sustainability of the effects of interventions used in digital self-monitoring in middle-aged and older patients with chronic diseases have not been adequately studied (Bartels et al., 2019).

Family health is “a resource at the level of the family unit that develops from the intersection of the health of each family member, their interactions and capacities, as well as the family’s physical, social, emotional, economic, and medical resources.” (Weiss-Laxer et al., 2020). Because members support and nurture each other at all stages of life in ways that other systems may not, families have unparalleled influence and resources to maintain health, prevent disease and predictive the attitudes of people towards palliative and hospice care (Medicine Io, Council NR, 2011; Robinson et al., 2017; Zhang et al., 2022). For most older adults, the family is the central aspect of the social environment (Logan and Spitze, 1996). This is especially true for health-related activities and concerns. For example, older adults are more likely to discuss health issues and symptoms with family members than with anyone else (Brody et al., 1983; Stoller et al., 1997).

Despite the centrality and economic value of families in creating health, little attention has been paid to measuring family health, in part because there are no validated measures of family health (Crandall et al., 2020). Therefore, the Chinese version of the Family Health Brief Scale (FHS-SF Chinese version), which involves multidimensional and interdisciplinary commonalities with good reliability and validity, was introduced by Chinese scholars Wang et al. and modified and adapted for the Chinese context, was used in this study (Wang et al., 2022). This scale divides family health into four dimensions: Family/social/emotional health processes, Family healthy lifestyle, Family health resources, and Family external social supports. Family/social/emotional health processes measure internal processes related to connectedness, communication, emotional security, satisfaction, and coping in the family environment; Family healthy lifestyle also addresses internal aspects of family health, including healthy behaviors and choices and habits that support health; Family health resources are divided into internal and external characteristics; internal resources include individual member health, family concerns, socioeconomic resources, and help-seeking effects external resources include access to resources and a family culture that trusts external resources; and family external social supports focus on external social supports available to families (Crandall et al., 2020).

Therefore, the purpose of this study is to investigate the mediating role of family health between frequency of smartphone use and self-management behavior among middle-aged and elderly patients with chronic diseases by constructing a mediating model of moderation, and whether this indirect relationship is modified by the solitary status of middle-aged and elderly Chinese patients with chronic diseases.

### Direct impact of smartphone use

The development of mHealth, refers to the use of mobile devices for medical and public health practices (World Health Organization, 2016), especially with the introduction to smartphones (Azevedo et al., 2015). Smartphones enable real-time health behavior monitoring and micro-level health behavior change (Payne et al., 2015), and various applications and health information in smartphones provide reference and basis for users’ behavioral decisions, disease prevention and healthcare (Guntuku et al., 2017; Chiu et al., 2019; Yang et al., 2020). For example, a randomized controlled trial implemented by Dale et al. (2015) in 123 patients with coronary heart disease in New Zealand found that the use of a mobile phone text messaging intervention in combination with a conventional intervention had a positive impact on increasing adherence and improving lifestyle in patients with coronary heart disease compared to a conventional chronic disease intervention alone (Dale et al., 2015). Results from a whole-group randomized controlled trial of middle-aged and older hypertensive patients in a community in Guangzhou, China suggest that a WeChat-based self-management intervention may be a feasible and effective option to help lower blood pressure and improve self-management in middle-aged and older hypertensive patients in Chinese communities (Li et al., 2019).

As such, We pose the following hypothesis:

*H1*: Smartphone use among middle-aged and older patients with chronic diseases will be positively associated with self-management behaviors.

### Indirect effect through family health

Family Health mediates the relationship between smartphones and chronic disease self-management behaviors. The use of smartphones by older Chinese people can improve relationships with their adult children and help maintain kinship ties (Lu and Kandilov, 2021). Not only that, but the use of mobile medical communication devices can also increase the perceived social support of older adults (Czaja et al., 2018). And, Emotional support from family members is strongly associated with the improvement of mobile Internet skill literacy and information literacy among middle-aged and older adults (Xiong and Zuo, 2019). The improvement of skill literacy and information literacy facilitates their screening and utilization of information, furthermore, the adoption of correct health behaviors and active disease self-management. What we also know is that family health is one of the strongest predictors of chronic disease self-management behaviors in older adults (Zhang et al., 2022). Families can assist with seeking information, providing recommendations, selecting and implementing actions, appraising implemented actions, and informing others about their experiences (Ligita et al., 2020). However, some studies have also found that certain behaviors of the family are detrimental to self-management in patients with chronic diseases (Clark and Nothwehr, 1997), it even has negative effects (Maclean, 1991).

As such, We pose the following hypothesis:

*H2*: There will be an indirect link between smartphone use and self-management behaviors in middle-aged and older patients with chronic diseases through family health.

### The moderating role of solitary state

**Figure 1:**
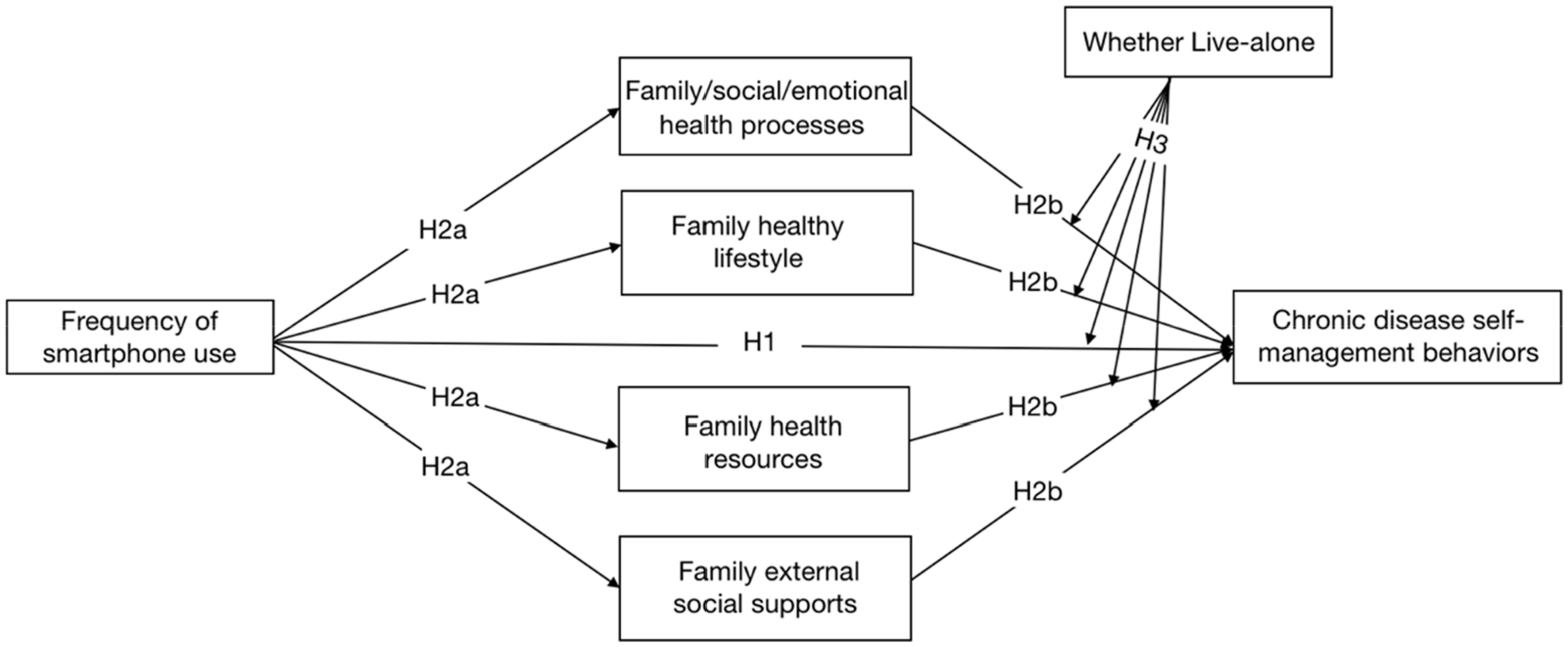
The hypothesized model. The chronic disease self-management behaviors variables we measured also included the three of these dimensions: “Exercise,” “Cognitive symptom management practices “, and “Communication with physicians.” due to the complexity of the model path, these three dimensions are not shown in the hypothetical model.

Previous studies have shown that living alone in middle-aged and older adults affects adherence to their medical regimens and health behavioral guidance and may even increase the risk of disease (Pedersen et al., 2016; Nam et al., 2021). Middle-aged and older people who live alone are those who do not live with their spouses or children and live alone. In addition, family emotional supports from family members are key factors influencing middle-aged and older people to live alone and maintain their health and promote recovery (Okkonen and Vanhanen, 2006; Xing and Rong, 2021). Previous scholarly research has found that Chinese middle-aged and older adults, especially those living alone, have a higher rate of unmet need for disease management due to the influence of family support compared to those non-living alone (Ji et al., 2021). Therefore, the final hypothesis is (Figure 1):

*H3*: The negative association of family health on self-management behaviors will be strongest when middle-aged and older patients with chronic diseases are living alone.

## Research methods

### Participants and procedures

The survey was carried out from July 10th, 2021 to September 15th, 2021, which involved the capital cities of 23 provinces and 5 autonomous regions, and 4 municipalities (Beijing, Tianjin, Shanghai, and Chongqing) all over China’s mainland. There were 120 cities, including the capital cities were directly included, and 2–6 other prefecture-level cities of each province and autonomous region chosen by random number table method. We recruited at least one investigator or a team of investigators in each city.

Based on the results of the “Seventh National Census in 2021” (Office of the Seventh National Population Census Leading Group of the State Council National Bureau of Statistics Office, 2021), we drew quota samples (quota attributes are gender, age, and urban–rural distribution) from the residents of the selected 120 cities, and verified the identity of the samples by enumerators recruited in these 120 cities so that the gender, age, and urban–rural distribution of the obtained samples were consistent with the demographic characteristics The gender, age, and urban–rural distribution of the obtained samples were generally consistent with the demographic characteristics.

With the help of the web-based Questionnaire Star platform, surveyors distributed questionnaires to the public one-on-one and face-to-face in their respective areas of responsibility. Respondents clicked on a link to obtain informed consent from the respondent and were asked to enter their questionnaire number to answer. If the respondent is capable of thinking but not mobile enough to answer the questionnaire, he/she is asked one-on-one by the surveyor and answers instead. A total of 11,709 questionnaires were distributed, and 11,031 valid questionnaires were returned, with an efficiency rate of 94.2%.

Information on socio demographic characteristics included gender, permanent residence, Solitary state, age range, number of chronic diseases, frequency of smartphone use in the past week, education level, Current way of bearing medical expenses, and family type (See Table 1). The inclusion criteria for respondents in this study were: age ≥ 12 years; the nationality of the People’s Republic of China; permanent residence in China (time spent away from home ≤1 month per year); voluntary participation in the study and completion of the informed consent form; the possibility of completing the online questionnaire on their own or with the help of the investigator; and understanding the meaning expressed in each entry of the questionnaire. Exclusion criteria were confusion or mental abnormality; being involved in other similar research projects; and unwillingness to cooperate.

**Table 1:** Summary of the demographic variable.

Variables	Number	Percent (%)
Gender		
Male	767	53.9
Female	657	46.1
Permanent residence		
Urban	949	66.6
Rural	475	33.4
Solitary state		
Live alone	142	10.0
Non-Live alone	1,282	90.0
Age range		
46–59	701	49.2
60–80	649	45.6
≥81	74	5.2
Number of chronic diseases		
One type of	872	61.2
More than one type of	552	38.8
Frequency of smartphone use in the past week		
Never use	259	18.2
≤1 day	98	6.9
2–3 days	158	11.1
4–5 days	197	13.8
6–7 days	712	50.0
Education level		
Primary school education and Lower education	445	31.3
Junior middle school education	299	21.0
Senior middle school education and vocational middle school education	282	19.8
Junior college	175	12.3
Bachelor degree, postgraduate degree and doctor’s degree	223	15.3
Current way of bearing medical expenses		
State expenditure	27	1.9
Resident medical insurance	764	53.7
Commercial medical insurance	25	1.8
Occupational medical insurance	442	31.0
At one’s own expense	166	11.7
Family types		
The stem family	393	27.6
Single-parent family	56	3.9
Single-person family	28	2.0
The nuclear family	516	36.2
Intergenerational family	40	2.8
Three generations	82	5.8
Conjugal family	275	19.3
DINK family	11	0.8
Other forms of family	23	1.6
Total	1,424	100

A total of 1,424 middle-aged and older patients with one type of and more than one type of chronic diseases aged 45 years or older were selected for this study, of whom the youngest age was 46 years and the oldest was 101 years. This research project was approved by the Institutional Review Board of Jinan University, Guangzhou, China (JNUKY-2021-018), and all respondents participated in the survey on a voluntary basis.

### Measures

#### The Chinese version of short-form of family health scale (FHS-SF Chinese version)

The Chinese version of short-form of Family Health Scale (FHS-SF Chinese version), modified and adapted for the Chinese context by Wang et al. (2022) was used. The questionnaire consisted of 10 questions, divided into four dimensions: Family/social/emotional health processes, family healthy lifestyle, Family health resources, and family external social supports. A five-point Likert scale was used, with 1 indicating “strongly disagree” and 5 indicating “strongly agree,” and the three questions on the family health resources dimension were scored in reverse, with 1 indicating “strongly agree” and 5 indicating “strongly disagree.” The Cronbach’s alpha coefficient for the Family Health Questionnaire in this study was 0.846, the Cronbach’s alpha coefficients for the four dimensions were 0.902, 0.858, 0.725, and 0.706, respectively. This indicates that the scales have good internal.

#### Chronic disease self-management behaviors scale (CDSBS-SF)

The Chronic Disease Self-Management Behaviors Scale, developed by the Center for Chronic Disease Education and Research at Stanford University, contains 15 items in three dimensions: “exercise,” “cognitive symptom management practices,” and “communication with physician,” The first dimension was assigned a score of 1 to 5 from “did not do” to “>3 h/week,” and the last two dimensions were assigned a score of 1 to 6 from “did not do” to “at all times,” respectively. The higher the score, the better the ability to self-manage chronic diseases. The Cronbach’s alpha coefficient for the Chronic Disease Self-Management Behaviors Scale in this study was 0.827. The Cronbach’s alpha coefficients for the three dimensions were 0.740, 0.811, and 0.783, respectively. This indicates that the scales have good internal consistency.

### Data analyses

#### Statistical analyses

After the data was recycled, the study was statistically analyzed using SPSS 26.0(SPSS, Chicago, IL, USA) and its significance level was set at 0.05. The Harman single factor test was first used to conduct factor analysis on the combination of so variables in the questionnaire, the results of the unrotated principal component analysis showed that there were two factors with characteristic roots greater than one, and the variance explained by the first factor was 34.780, which was below the critical criterion of (40%), indicating that there was no significant common method bias problem in this study. In this study, Pearson analysis was used to examine the correlations of all variables. Then the multicollinearity problem between the variables was checked and the results showed that the VIF values were all less than 5 (Alin, 2010), so there was no multicollinearity problem between the variables. Finally, model 4 of SPSS Macro PROCESS 3.1 was used to test the mediating role of family health between the frequency of smartphone use and chronic disease self-management behaviors by generating bias-corrected bootstrap confidence intervals (using a bootstrap sample of 5,000), Using the method and software described in Model 4, Model 14 was selected to test the mediation model of conditioning.

### Control variables

First of all, through literature review, we found differences in self-management behaviors among middle-aged and older patients with chronic diseases by gender (Theeke et al., 2019), family economic income (Gonzalez-Zacarias et al., 2016; Kneipp et al., 2019), and Permanent Residence (Callahan, 2021). In addition, the Pearson correlation results showed that gender, family economic income, and usual residence were significantly correlated with the variables surveyed (see Table 2). Thus, they were treated as control variables in all analyses afterward. Gender was coded as male = 0, female = 1; the permanent residence was coded as urban = 0, rural = 1, and family economic income as a continuous variable.

**Table 2:** Key variables and Pearson correlation coefficients of all variables.

	M ± SD	1	2	3	4	5	6	7	8	9	10	11	12
1.FOSU	3.71 ± 1.562	1.000											
2.FSEHP	12.16 ± 2.373	0.233**	1.000										
3.FHL	8.17 ± 1.622	0.215**	0.870**	1.000									
4.FHR	10.74 ± 2.873	0.103**	0.258**	0.233**	1.000								
5.FESS	7.66 ± 1.581	0.176**	0.590**	0.589**	0.165**	1.000							
6.CDSB	34.86 ± 9.466	0.267**	0.114**	0.107**	−0.085**	0.124**	1.000						
7.Exercise	11.26 ± 4.425	0.220**	0.041	0.031	−0.100**	0.045	0.731**	1.000					
8.CSMP	14.19 ± 4.779	0.189**	0.066*	0.065*	−0.110**	0.077**	0.816**	0.342**	1.000				
9.CWP	9.41 ± 3.386	0.194**	0.172**	0.167*	0.047	0.178**	0.689**	0.253**	0.422**	1.000			
10.Gender		0.070**	−0.026	−0.025	−0.021	−0.008	0.089**	0.122**	0.052*	−0.017	1.000		
11.FEI		0.234**	0.072**	0.048	0.098**	0.066*	0.165**	0.202**	0.100**	0.056*	0.033	1.000	
12.PR		−0.279**	−0.090**	−0.074**	−0.046	−0.050	−0.142**	−0.157**	−0.098**	−0.053*	0.009	−0.311**	1.000

*N* = 1,424; *p < 0.05, **p < 0.01. FOSU = Frequency of smartphone use; FSEHP = Family/social/emotional health processes; FHL = Family Healthy Lifestyle; FHR = Family health resources; FESS=Family external social supports; CDSB = Chronic disease self-management behaviors; CSMP = Cognitive symptom management practices; CWP=Communication with physicians; FEI=Family economic income; PR = Permanent Residence.

## Results

### Preliminary analyses

The relationship between the frequency of smartphone use, family health, and chronic disease self-management behaviors is shown in Table 2. The frequency of smartphone use was significantly and positively associated with family health and chronic disease self-management behaviors (*p* < 0.01); The dimension of family health resources in family health was significantly and negatively associated with chronic disease self-management behaviors (*p* < 0.01), Family/social/emotional health processes, family healthy lifestyle and family external social supports dimensions of family health were significantly and positively associated with chronic disease self-management behaviors (*p* < 0.01). All variables were significantly correlated with each other (*p* < 0.01, *p* < 0.05) as shown in Table 2.

### Testing for the mediation effects of family health

As shown in Table 3, the results of the mediation analysis showed that frequency of smartphone use indirectly had a negative predictive effect on the self-management behaviors of middle-aged and older patients with chronic diseases through the family health resources dimension (*β* = −0.0758, CI: −0.1402, −0.0236), and indirectly through the family external social supports dimension of the family health to positively predict the self-management behaviors of middle-aged and older patients with chronic diseases (*β* = 0.0721, CI: 0.0141, 0.1458). Frequency of smartphone use negatively predicts exercise and cognitive symptom management practices indirectly through the family health resources dimension in middle-aged and older patients with chronic diseases (*β* = −0.0344, CI: −0.0652, −0.0102; *β* = −0.0401, CI:-0.0725, −0.0138),and indirectly, through the family external social supports dimension of the family health, had a positive predictive effect on the physician communication aspects of middle-aged and older patients with chronic diseases (*β* = 0.0376, CI: 0.0116, 0.0705).

**Table 3:** Bootstrapping the conditional indirect effect and 95% confidence interval (CI) for the moderated mediation model.

Indirect path	Indirect Effect	SE	LLCI	ULCI
FOSU→ FHR→ CDSB	−0.0758	0.0296	−0.1402	−0.0236
FOSU → FESS→ CDSB	0.0721	0.0340	0.0141	0.1458
FOSU → FHR→ Exercise	−0.0344	0.0138	−0.0652	−0.0102
FOSU → FHR→ CSMP	−0.0401	0.0151	−0.0725	−0.0138
FOSU → FESS→ Exercise	0.0376	0.0139	0.0116	0.0705

*N* = 1,424; Bootstrap sample size = 5,000; LL = low limit; CI = confidence interval; UL = upper limit. FOSU = Frequency of smartphone use; FHR = Family health resources; FESS = Family external social supports; CDSB = Chronic disease self-management behaviors; CSMP = Cognitive symptom management practices.

### Testing for the moderated mediation effects of whether live-alone

**Figure 2:**
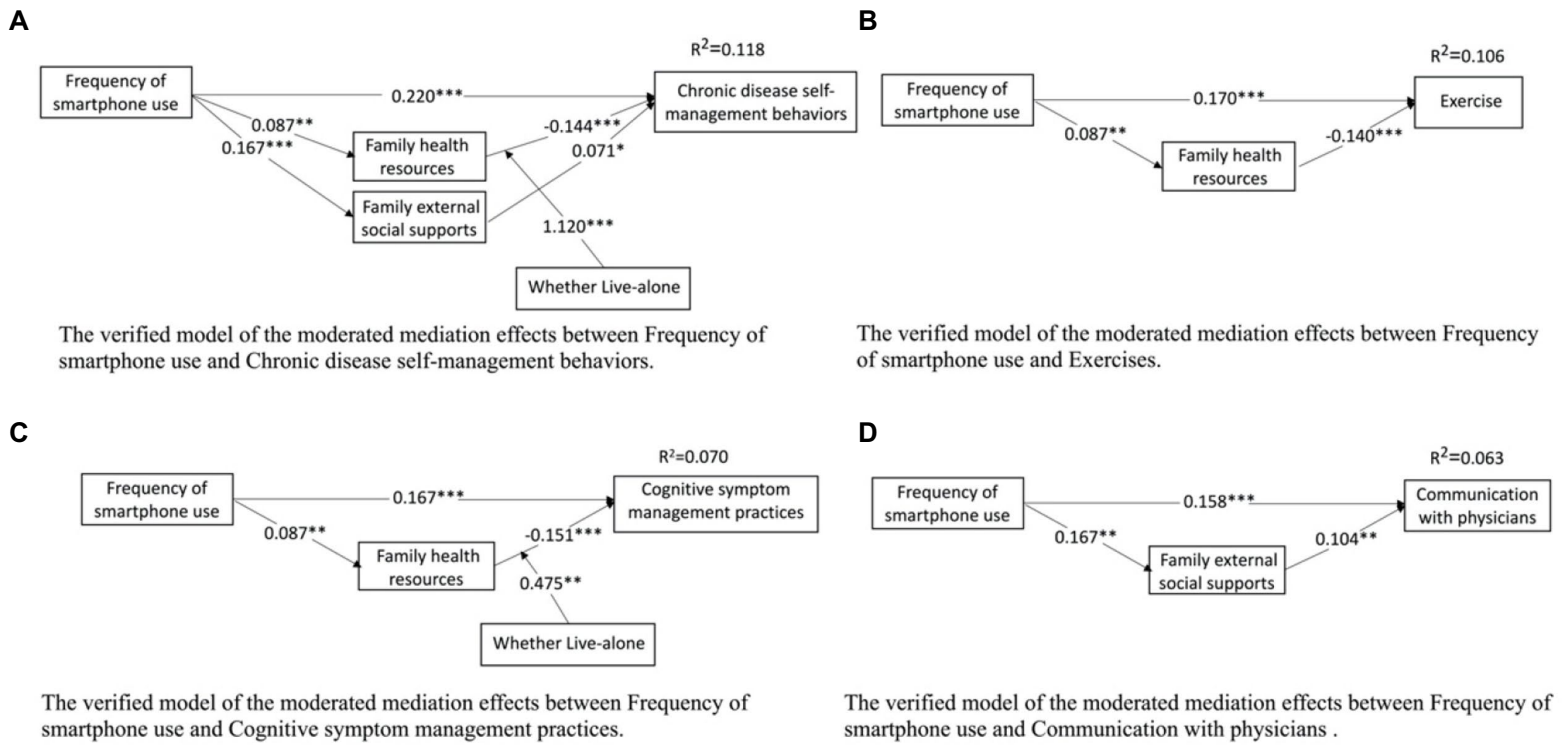
Mediated moderating effect validation models. The verified model of the moderated mediation effects between Frequency of smartphone use and Chronic disease self-management behaviors, and between Frequency of smartphone use and Exercise, Cognitive symptom management practices and Communication with physicians. *p < 0.05, **p < 0.01, ***p < 0.001.

**Figure 3:**
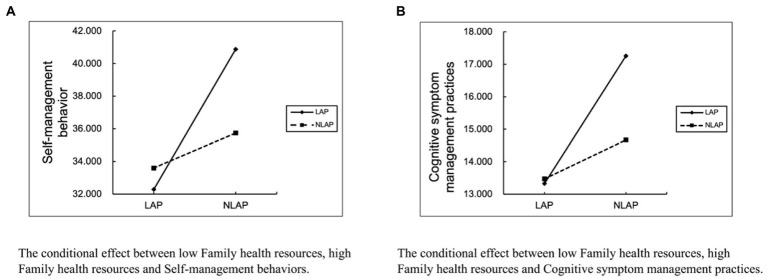
“Whether live-alone” moderating effect slope plots. The conditional effect between low family health resources, high family health resources **(A)** Self-management behaviors and **(B)** Cognitive symptom management practices. LAP = Live alone patients; NLAP = Not live alone patients.

The results of the tests for moderating mediating effects using the PROCESS 3.3 plug-in in SPSS 26.0 are shown in Tables 4, 5. Frequency of smartphone use was a direct positive predictor of self-management behaviors and cognitive symptom management practice dimensions, after controlling for gender, family income and Permanent Residence (*β* = 1.3009, *β* = 0.4951 *p* < 0.001; See Table 4), and indirect predictions were made through the family health resources dimension in family health (live alone, *β* = −0.2395, CI: −0.4574, −0.0661; *β* = −0.1095, CI: −0.1961, −0.0378; not live-alone, *β* = −0.0599, CI: −0.1164, −0.0172; *β* = −0.0334, CI: −0.0633, −0.0102), rather than through the Family/social/emotional health processes dimension and the life and family external social supports dimension (live alone, *β* = 0.1818, CI: −0.1794, 0.5648; *β* = −0.0796, CI: −0.4874, 0.2821; *β* = 0.1026, CI: −0.1061, 0.3278; *β* = 0.0751, CI: −0.1211, 0.2784; *β* = −0.0459, CI: −0.2393, 0.1256; *β* = 0.1256, CI: −0.0621, 0.1570; not live-alone, *β* = 0.0443, CI: −0.1053, 0.1981; *β* = 0.0354, CI: −0.1018, 0.1745; *β* = 0.0094, CI: −0.0684, 0.0876; *β* = 0.0221, CI: −0.0480, 0.0945; *β* = 0.0224, CI: −0.0106, 0.0584; see Table 4). That is, the family health resources dimension of family health partially mediates the relationship between family health and self-management behaviors. The interaction of the family health resources dimension and “Whether Live-alone “had a significant effect on self-management behaviors and cognitive symptom management practice dimensions (FHR × WL-A, *β* = −1.1210; *β* = −0.4751, *p* < 0.001; *p* < 0.01, CI: −1.7053, −0.5367; CI: −0.7784, −0.5367; see Table 4). The validated regulation model was shown in Figure 2A. To further understand the moderating effect of pathways between family health resources dimensions and self-management behaviors among middle-aged and older chronic disease patients live alone. The indirect effects of moderation were analyzed in two aspects of chronic diseases in middle-aged and older patients live alone (see Figure 2). Family health resources were significantly and negatively associated with self-management behaviors and cognitive symptom management practice dimensions among middle-aged and older chronic disease patients live alone, but not among non-live alone chronic disease patients. Slope plots (see Figure 3) suggest that the negative effect of the family health resources dimension on self-management behaviors and cognitive symptom management practice dimensions is greater in a population of middle-aged and older chronic disease patients live alone.

**Table 4:** The moderated mediation analyses.

Outcome variable	Independent variables	*β*	SE	*t*	*p*	LLCI	ULCI
CDSB	Constant	28.2824***	0.9831	28.7697	<0.001	26.3540	30.2108
	PR	−1.0504	0.5409	−1.9419	0.0523	−2.1114	0.0107
	FEI	1.2259***	0.3020	4.0586	<0.001	0.6334	1.8184
	Gender	1.2974**	0.4748	2.7327	<0.01	2.2287	0.3661
	FOSU	1.3009***	0.1643	7.9157	<0.001	0.9785	1.6232
	FSEHP	0.1686	0.2099	0.8032	0.4220	−0.2431	0.5802
	FHL	0.1075	0.3014	0.3565	0.7215	−0.4839	0.6988
	FHR	−0.4859***	0.0853	−5.6952	<0.001	−0.6532	−0.3185
	FESS	0.4075*	0.1892	2.1539	<0.05	0.0364	0.7786
	WL-A	0.6522	0.3289	1.9829	0.0477	0.0198	1.2833
	FSEHP*WL-A	0.3995	0.6164	0.6482	0.5169	−0.8095	1.6086
	FHL*WL-A	−0.5167	0.8725	−0.5922	0.5538	−2.2282	1.1948
	FHR*WL-A	−1.1210***	0.2978	−3.7638	<0.001	−1.7053	−0.5367
	FESS*WL-A	0.2010	0.6104	0.3293	0.7420	−0.9965	1.3984
	*R* ^2^	0.1271***					
	F	15.7899					
CSMP	constant	11.8346***	0.5104	23.1889	<0.001	10.8335	12.8357
	PR	−0.4109	0.2808	−1.4633	0.1436	−0.9617	0.1399
	FEI	0.3722*	0.1568	2.3737	<0.05	0.0646	0.6798
	Gender	0.3480	0.2465	1.4121	0.1582	−0.8315	0.1355
	FOSU	0.4951***	0.0853	5.8034	<0.001	0.3278	0.6625
	FSEHP	0.0464	0.1089	0.4255	0.6705	−0.1674	0.2601
	FHL	0.0689	0.1565	0.4402	0.6599	−0.2381	0.3759
	FHR	−0.2562***	0.0443	−5.7842	<0.001	−0.3431	−0.1693
	FESS	0.1385	0.0982	1.4100	0.1587	−0.0542	0.3311
	WL-A	1.2224**	0.4292	2.8485	<0.01	0.3806	2.0643
	FSEHP*WL-A	0.1910	0 0.3200	0.5969	0.5506	−0.4367	0.8187
	FHL*WL-A	−0.3058	0.4529	−0.6752	0.4997	−1.1943	0.5827
	FHR*WL-A	−0.4751**	0.1546	−3.0724	<0.01	−0.7784	−0.1717
	FESS*WL-A	0.1032	0.3169	0.3258	0.7446	−0.5184	0.7249
	*R* ^2^	0.0769***					
	*F*	9.0294					

*N* = 1,424; *p < 0.05, **p < 0.01, ***p < 0.001, PR = Permanent Residence; FEI = Family economic income; FOSU = Frequency of smartphone use; FSEHP = Family/social/emotional health processes; FHR = Family health resources; FESS = Family external social supports; WL-A = Whether Live-alone; CDSB = Chronic disease self-management behaviors; CSMP = Cognitive symptom management practices.

**Table 5:** Bootstrapping the conditional indirect effect and 95% confidence interval (CI) for the moderated mediation model.

Path	Whether live-alone	Indirect effect	SE	LLCI	ULCI
FOSU→FSEHP→CDSB	Non-live alone	0.0443^b^	0.0772	−0.1053	0.1981
	Live alone	0.1818^b^	0.1885	−0.1794	0.5648
FOSU→FHL → CDSB	Non-live alone	0.0354^b^	0.0703	−0.1018	0.1745
	Live alone	−0.0796^b^	0.1945	−0.4874	0.2821
FOSU→FHR → CDSB	Non-live alone	−0.0599^a^	0.0256	−0.1164	−0.0172
	Live alone	−0.2395^a^	0.1000	−0.4574	−0.0661
FOSU→FESS→CDSB	Non-live alone	0.0676^a^	0.0366	0.0008	0.1478
	Live alone	0.1026^b^	0.1074	−0.1061	0.3278
FOSU→FSEHP→CSMP	Non-live alone	0.0094^b^	0.0397	0.0684	0.0876
	Live alone	0.0751^b^	0.1012	−0.1211	0.2784
FOSU→FHL → CSMP	Non-live alone	0.0221^b^	0.0368	−0.0480	0.0945
	Live alone	−0.0459^b^	0.0924	−0.2393	0.1256
FOSU→FHR → CSMP	Non-live alone	−0.0334^a^	0.0135	−0.0633	−0.0102
	Live alone	−0.1095^a^	0.0408	−0.1961	−0.0378
FOSU→FESS→CSMP	Non-Live alone	0.0224^b^	0.0175	−0.0106	0.0584
	Live alone	0.0404^b^	0.0538	−0.0621	0.1570

*N* = 1,424; Bootstrap sample size = 5,000; LL = low limit; CI = confidence interval; UL = upper limit; FOSU = Frequency of smartphone use; FSEHP = Family/social/emotional health processes; FHL = Family healthy lifestyle; FHR = Family health resources; FESS = Family external social supports; CDSB = Chronic disease self-management behaviors; CSMP = Cognitive symptom management practices; CWP = Communication with physicians.

a Empirical 95% confidence interval does not overlap with zero.

b Empirical 95% confidence interval overlaps with zero.

## Discussion

Our investigation focused on the frequency of smartphone use, chronic disease self-management behaviors, and family health. By constructing a moderated mediation model, we found that the frequency of smartphone use directly and indirectly predicted the self-management behaviors of middle-aged and older patients with chronic diseases through family health in terms of family health resources and external social support at family. In addition, “Whether Living-alone “moderated the relationship between the family health resources dimension of family health and perceived symptom management practices of self-management behaviors in middle-aged and older patients with chronic diseases.

Firstly, this study found a positive correlation between the frequency of smartphone use and both the family health dimension of family health and the external family social support dimension, suggesting that the more frequent smartphone exposure among middle-aged and older patients with chronic diseases, the more family health resources and external family social support they have. This finding of the present study corresponds to the previous findings that a pilot study of smartphone medication reminder apps for middle-aged and older adults with chronic conditions suggests that their family members may view their smartphone medication reminder apps positively in primary care settings (Park et al., 2017). The positive association for social support outside the home can be explained by the fact that smartphone exposure and use is an important way to improve their social connection to the outside world (Chen and Schulz, 2016). Through access to smartphones, older adults are able to maintain and strengthen their social connections, thereby increasing their externally available social resources (Quan-Haase et al., 2017).

We then found that the frequency of smartphone use was significantly and positively associated with the self-management behaviors of middle-aged and older patients with chronic diseases, indicating that the more frequently middle-aged and older patients with chronic diseases had smartphone use, the better the self-management behaviors (exercise, cognitive symptom management practices, and communication with physicians). Previous scholars have suggested that the use of smartphones for chronic disease interventions may help support self-management of long-term illness in patients with chronic diseases (De Jongh et al., 2012; Steinert et al., 2020). Because the development, input and use of mobile health programs in smartphones is an effective and cost-efficient way to improve the treatment of patients with chronic diseases in self-management of chronic diseases (Fitzner et al., 2014). For patients with chronic illnesses that require long-term suffering, mHealth applications may offer the convenience of providing information such as medication reminders, treatment adjustments, or supportive messages *via* SMS to patients with chronic illnesses (Kwon et al., 2020), and supported patient empowerment, participants advocated for themselves by becoming the leader of their health, especially when communicating with their physician (Schmaderer et al., 2021). In addition, the use of social media and physical activity apps may also can explain. Social media platforms in smartphones can provide the experience of others (van Berkel et al., 2015). Middle aged and elderly patients with chronic diseases can obtain social support and advice from other people with similar health related experiences, to help them manage their health. Chopik (2016) found that higher social technology use was associated with less chronic disease in older adults (Chopik, 2016). And patients with chronic diseases have access to a self-management strategy for physical activity apps in smartphones (Bonn et al., 2018). The value of the WeChat app in chronic disease management in China has been proven (Chen et al., 2020). Perhaps the WeChat Sports app in smartphones is also a key potential factor influencing self-management behaviors in middle-aged and elderly patients with chronic diseases. The WeChat Sports app has automatic tracking and daily ranking features can help Chinese middle-aged and elderly people with chronic diseases record and increase their daily walking steps.

Secondly, family health and self-management behaviors of middle-aged and older patients with chronic diseases were negatively associated, as evidenced by the negative effect of the family health resources dimension of family health on the exercise and cognitive symptom management practices of middle-aged and older patients with chronic diseases. This suggests that the more family health resources available, the poorer the exercise and cognitive symptom management practices of middle-aged and older patients with chronic conditions. The reason may be that other members of the family are healthy, unaware of the chronic disease, and adopt unsupportive behaviors. Family members may undermine self-management in people with chronic disease by promoting unhealthy behaviors, discouraging healthy behaviors, or providing advice that conflicts with self-management advice, these effects were exemplified by El-Kebbi et al. (1996) in a study of social barriers to dietary adherence in African American adults with diabetes. For example, nagging, denial of the severity of the disease, dietary advice that conflicts with recommended diabetes management (El-Kebbi et al., 1996; Maillet et al., 1996),and the reluctance of family members to adjust their diets makes them develop unhealthy eating habits (Mei et al., 2022). All of these factors may have a negative impact on the self-management of patients with chronic diseases.

In addition, an interesting finding of this study was that smartphone exposure had both positive and negative effects on chronic disease self-management behaviors under the mediating effect of family health. However, only a small body of literature has previously addressed the possibility that family health may have a positive or negative impact, either directly or indirectly, on chronic disease self-management behaviors (Gallant et al., 2007). Specifically, the family health resources dimension and the external social support dimension of family health moderated the effect of frequency of smartphone use on self-management behaviors of middle-aged and older patients with chronic diseases. That is, the more frequent smartphone use among middle-aged and older patients with chronic diseases, the more their family health resources and family external social supports, and the more family health resources the less active exercise and cognitive symptom management practices among middle-aged and older patients with chronic diseases. And, the more family external social supports, the more active the middle-aged and older chronic disease patients were in communicating with their physicians.

Finally, this study demonstrates that “whether living-alone “moderates the relationship between the family health resources dimension of family health and self-management behaviors and the cognitive symptom management practice dimension of self-management behaviors in middle-aged and older chronic patients. In other words, the moderating effect of living alone with chronic diseases on the relationship between family health resources and self-management behaviors of middle-aged and older patients with chronic diseases and the relationship between family health resources and the cognitive symptom management practice dimension of self-management behaviors was more significant than that of non-living alone middle-aged and older patients with chronic diseases. This finding is consistent with differences in chronic disease self-management behaviors between the living-alone and not living-alone groups (Mingo et al., 2015). Further, a seven-year qualitative study conducted in the United States may better explain the finding that people who live alone tend to be more socially and civically active, enjoy richer and more varied life experiences, and may be more positive about health management (Klinenberg, 2013). When adults living alone have less contact with their families, they seek other sources of support from neighbors, religious groups or the community (Kao et al., 2013; Yi and Hwang, 2015).

## Limitations and implications

There are still some limitations that need to be addressed in future studies. First, current cross-sectional studies have not been able to determine a causal relationship between frequency of smartphone use and chronic disease self-management behaviors. More in-depth results will require further exploration of future longitudinal approaches. Second, although this study explored the frequency of smartphone use as a positive predictor of self-management behaviors in middle-aged and older patients with chronic diseases, it remains to be investigated which programs in smartphones are most effective.

Despite the limitations, the present findings have important practical implications. An important research implication of this study may be that it is the first study to examine the mechanisms between the frequency of smartphone use, family health, and chronic disease self-management behaviors. The frequency of smartphone use can directly and indirectly predict the self-management behaviors of middle-aged and older patients with chronic diseases through the family health resources dimension and family external social supports dimension in family health. The indirect association between the family health resources dimension and chronic disease self-management behaviors, and the cognitive symptom management practice dimension of chronic disease self-management behaviors, was moderated by “whether living-alone.” Studies of family health in middle-aged and older adults with chronic disease may help build a knowledge base that can be used to map phenotypes, and identifying phenotypes associated with family health may help investigate the underlying mechanisms underlying self-management behaviors for chronic disease.

## Conclusion

The current study indicates that the frequency of smartphone use was the strongest positive predictor of self-management behaviors among middle-aged and older patients with chronic diseases. Family health partially mediated the relationship between frequency of smartphone use and chronic disease self-management behaviors, but not all dimensions. Whether live-alone played a moderating role in the negative association between family health resources and self-management behaviors among middle-aged and older patients with chronic diseases. Based on the results of our study, it helps to develop interventions. By improving the self-management behaviors of middle-aged and older chronic patients, the overall health status of chronic patients can be improved.

## Data availability statement

The raw data supporting the conclusions of this article will be made available by the authors, without undue reservation. For data acquisition contact E-mail: bjmuwuyibo@outlook.com.

## Ethics statement

The studies involving human participants were reviewed and approved by The Institutional Review Committee of Ji’nan University, Guangzhou, China (JNUKY-2021-018). The patients/participants provided their written informed consent to participate in this study.

## Author contributions

FG and ZL: conceptualization, formal analysis, and writing—original draft preparation. ZL: methodology and software. FG, ZL, and YW: validation. YW: investigation and resources. YY: data curation. YW, HM, YY, ZH, JL, WW, JT, and WW: review and editing. ZL: visualization. XS: supervision. YW: project administration. FG: funding acquisition. All authors contributed to the article and approved the submitted version.

## Funding

This study was supported by Hunan Provincial Social Science Achievement Review Committee Project in 2018: Study on the Media View of the North American School of Media Environment in the Perspective of the History of Communication Thought (Project No. XSP18YBC139), National Social Science Foundation Project in 2019: Study on the Media Philosophy of the North American School of Media Environment (Project No. 19BZX036) and the Cultural Research Center of Countries along the “Southwest Silk Road” of the Ethnic Affairs Commission of the People’s Republic of China (Jishou University).

## Conflict of interest

The authors declare that the research was conducted in the absence of any commercial or financial relationships that could be construed as a potential conflict of interest.

## Publisher’s note

All claims expressed in this article are solely those of the authors and do not necessarily represent those of their affiliated organizations, or those of the publisher, the editors and the reviewers. Any product that may be evaluated in this article, or claim that may be made by its manufacturer, is not guaranteed or endorsed by the publisher.

## Abbreviations

SBAMAOPWCD: self-management behaviors among middle-aged and older patients with chronic diseases
FH: Family Healthy
FOSU: Frequency of smartphone use
FSEHP: Family/social/emotional health processes
FHL: Family healthy lifestyle
FHR: Family health resources
FESS: Family external social supports
CDSB: Chronic disease self-management behaviors
CSMP: Cognitive symptom management practices
CWP: Communication with physicians
